# Effects of Quinine on the Glycaemic Response to, and Gastric Emptying of, a Mixed-Nutrient Drink in Females and Males

**DOI:** 10.3390/nu15163584

**Published:** 2023-08-15

**Authors:** Peyman Rezaie, Vida Bitarafan, Braden David Rose, Kylie Lange, Zinat Mohammadpour, Jens Frederik Rehfeld, Michael Horowitz, Christine Feinle-Bisset

**Affiliations:** 1 Adelaide Medical School, University of Adelaide, Adelaide, SA 5005, Australia; 2 Centre of Research Excellence in Translating Nutritional Science to Good Health, University of Adelaide, Adelaide, SA 5005, Australia; 3 Nutrition, Diabetes and Gut Health, Lifelong Health Theme, South Australian Health and Medical Research Institute, Adelaide, SA 5001, Australia; 4 School of Biomedicine, University of Adelaide, Adelaide, SA 5005, Australia; 5 Department of Clinical Biochemistry, Rigshospitalet, University of Copenhagen, DK-2100 Copenhagen, Denmark; 6 Endocrine and Metabolic Unit, Royal Adelaide Hospital, Adelaide, SA 5005, Australia

**Keywords:** postprandial blood glucose, bitter taste, gut functions, gastrointestinal hormones, pancreatic hormones, human

## Abstract

Intraduodenal quinine, in the dose of 600 mg, stimulates glucagon-like peptide-1 (GLP-1), cholecystokinin and insulin; slows gastric emptying (GE); and lowers post-meal glucose in men. Oral sensitivity to bitter substances may be greater in women than men. We, accordingly, evaluated the dose-related effects of quinine on GE, and the glycaemic responses to, a mixed-nutrient drink in females, and compared the effects of the higher dose with those in males. A total of 13 female and 13 male healthy volunteers received quinine-hydrochloride (600 mg (‘QHCl-600’) or 300 mg (‘QHCl-300’, females only) or control (‘C’), intraduodenally (10 mL bolus) 30 min before a drink (500 kcal, 74 g carbohydrates). Plasma glucose, insulin, *C*-peptide, GLP-1, glucose-dependent insulinotropic polypeptide (GIP) and cholecystokinin were measured at baseline, for 30 min after quinine alone, and then for 2 h post-drink. GE was measured by ^13^C-acetate breath-test. QHCl-600 alone stimulated insulin, *C*-peptide and GLP-1 secretion compared to C. Post-drink, QHCl-600 reduced plasma glucose, stimulated *C*-peptide and GLP-1, and increased the *C*-peptide/glucose ratio and oral disposition index, while cholecystokinin and GIP were less, in females and males. QHCl-600 also slowed GE compared to C in males and compared to QHCl-300 in females (*p* < 0.05). QHCl-300 reduced post-meal glucose concentrations and increased the *C*-peptide/glucose ratio, compared to C (*p* < 0.05). Magnitudes of glucose lowering and increase in *C*-peptide/glucose ratio by QHCl-600 were greater in females than males (*p* < 0.05). We conclude that quinine modulates glucoregulatory functions, associated with glucose lowering in healthy males and females. However, glucose lowering appears to be greater in females than males, without apparent differential effects on GI functions.

## 1. Introduction

The upper gastrointestinal (GI) tract plays a critical role in the regulation of postprandial blood glucose concentrations [1,2]. The arrival of nutrients in the small intestine stimulates GI hormones, including glucagon-like peptide-1 (GLP-1), glucose-dependent insulinotropic polypeptide (GIP) and cholecystokinin (CCK) [3,4,5,6]. GLP-1 and CCK potently slow gastric emptying [1,7], which reduces the rate of delivery and, hence, the absorption of nutrients, including carbohydrates, and is a key determinant of post-meal blood glucose concentrations [8]. GLP-1 also stimulates insulin, and suppresses glucagon, in a glucose-dependent fashion [9], although its primary postprandial glucose-lowering action is via slowing of gastric emptying [10].

The intravenous administration of quinine (thus, bypassing the GI tract) for the treatment of malaria, in a dose of ~500 mg, has long been known to trigger hypoglycaemia in some patients, due to the potent stimulation of insulin [11,12]. In contrast, in healthy volunteers, acute intravenous infusion of the same dose has been reported to lower fasting blood glucose but does not appear to be associated with hypoglycaemia [13]. There has been increased interest in the GI effects of bitter substances, reflecting preclinical observations in both cell lines and experimental animals that show they stimulate the secretion of CCK and GLP-1, as well as other GI hormones potently by activating bitter taste receptors located on enteroendocrine cells [14,15,16,17], although information relating to GIP is limited [18]. The bitter substance, quinine, also stimulates plasma CCK and GLP-1 in healthy men [19,20,21,22]. For example, the intraduodenal (ID) administration of quinine hydrochloride (QHCl), in a dose of 600 mg (corresponding to ~500 mg quinine, often given intravenously in the treatment of malaria), stimulates CCK and GLP-1 in healthy men [20]. The magnitude of this stimulation by quinine is greater following ID than intragastric (IG) administration [20], which is indicative of the importance of the interaction of quinine with small intestinal bitter receptors. QHCl also stimulates insulin secretion and slows gastric emptying (the latter probably mediated, at least in part, by CCK and GLP-1), which, along with the stimulation of GLP-1, may underlie quinine-induced glucose lowering [21]. Indeed, in our recent study, QHCl, in a dose of 600 mg, administered either ID 30 min or IG 60 min before a mixed-nutrient drink stimulated insulin, slowed gastric emptying and reduced blood glucose in healthy men [19]. Moreover, the effect of IG quinine in men was dose-related [22]. Of interest, in the earlier study [22], in which QHCl was administered IG 30 min before a mixed-nutrient drink, quinine did not affect gastric emptying of the drink, which is in contrast to the slowing evident when QHCl was administered IG 60 min before a mixed-nutrient drink, providing further evidence of the importance of small intestinal exposure.

Oral sensitivity to 6-n-propylthiouracil, a bitter compound, has been reported to be greater in women than men [23,24]. It is, accordingly, of interest that we have recently found that ID QHCl, given in a dose of 600 mg, stimulates insulin and lowers fasting blood glucose more in women than men [20]. The glucose-lowering effect of quinine in females has only been evaluated in the fasted state; therefore, key questions about the dose-related effects of quinine on the glycaemic response to a meal, and whether females are more sensitive than males, remain unanswered.

The aims of this study were to determine (1) the effects of ID administration of two doses of QHCl (300 mg and 600 mg) on plasma glucose, pancreatic (i.e., insulin, *C*-peptide (reflecting insulin secretion)) and gut (i.e., GLP-1, GIP and CCK) hormones, and the gastric emptying of a mixed-nutrient drink in females and (2) the comparative effects of the higher dose of quinine in females and males.

## 2. Materials and Methods

### 2.1. Study Participants

**Figure 1:**
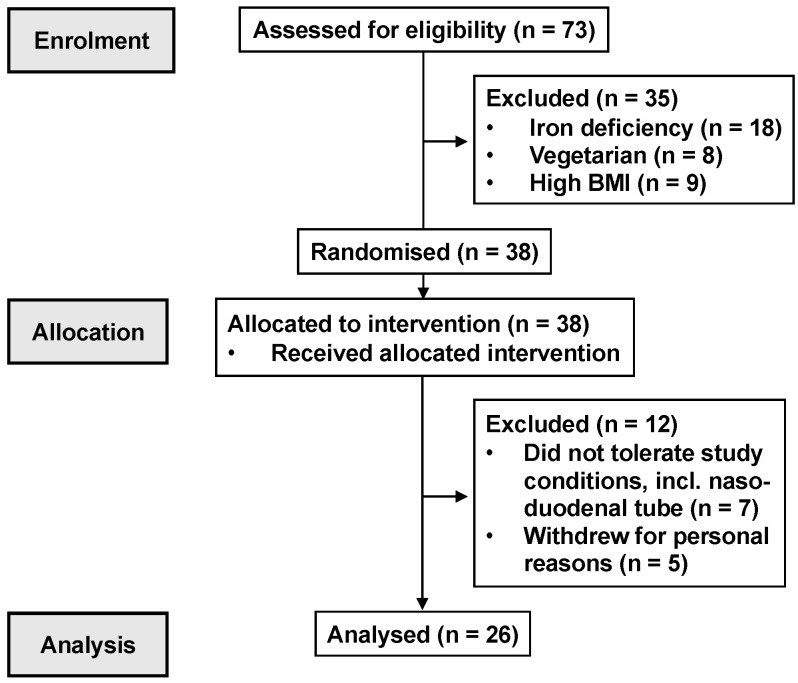
CONSORT participant flow diagram.

13 healthy, premenopausal females (mean age: 27 ± 2.1 years, body mass index (BMI): 22.3 ± 0.5 kg/m^2^, body weight: 59 ± 2 kg) and 13 healthy males (mean age: 26 ± 2 years, BMI: 22.7 ± 0.5 kg/m^2^, body weight: 71 ± 2 kg) participated in this study (Figure 1). The number of participants allowed for the detection of a 1.0 mmol/L difference in peak plasma glucose between the 600 mg quinine dose and control, with an SD of 0.9 mmol/L, α = 0.05 and a power of 80%. Participants were recruited through flyers placed around local universities. They were screened before their inclusion to exclude a history of GI disease or surgery; GI symptoms; smokers; individuals with overweight/obesity (BMI > 25 kg/m^2^); an alcohol consumption of >20 g/day on >5 days/week; use of medication known to affect appetite, energy intake or GI function; unstable body weight (≥5% change over the 3 months prior to participation); high-performance athletes; and restrained eaters (score >12 on the restrained eating component of the 3-factor eating questionnaire) [25]. Oral detection thresholds for QHCl were quantified using the ascending-series 3-alternative forced-choice technique [22,26] to ensure that all participants detected QHCl.

The study protocol was approved by the Human Research Ethics Committee of the Central Adelaide Local Health Network and was performed in accordance with the Declaration of Helsinki. All participants provided written, informed consent before their inclusion, and after enrolment, each was assigned to a treatment order of balanced randomisation that was generated using an online tool (www.randomization.com) by a research officer who was not involved in the data analysis. The study was registered as a clinical trial with the Australian and New Zealand Clinical Trials Registry (www.anzctr.org.au; ACTRN12620001139965; ACTRN12619001269123).

### 2.2. Study Outline

The double-blind, randomised cross-over study evaluated the effects of ID administration of QHCl, in doses of 300 and 600 mg, on plasma glucose, pancreatic (i.e., insulin and *C*-peptide) and gut (i.e., GLP-1, GIP and CCK) hormones and the gastric emptying of a mixed-nutrient drink in healthy, lean women, and compared the effects of the higher (and, therefore, most likely to be more potent) dose of QHCl in males and females. Plasma glucose was the primary outcome, while plasma insulin, *C*-peptide, GLP-1, GIP and CCK and gastric emptying were secondary outcomes. The 600-mg dose of QHCl was selected based on our previous studies, in which ID administration stimulated plasma GLP-1, insulin and *C*-peptide, and reduced fasting blood glucose [20]. A dose of 300 mg was chosen to determine whether a lower dose would also be effective in females. ID administration was selected because of the evidence of greater GI effects of ID than IG administration [20], and the timing of QHCl administration, 30 min before the mixed-nutrient drink, was based on our findings that the effects of ID quinine on stimulating plasma insulin and pyloric pressures were maximal at around that time [20].

### 2.3. Preparation of Treatments

The QHCl treatments were prepared by dissolving 300 mg or 600 mg of QHCl (Sinkona Indonesia Lestari, Subang, West Java, Indonesia) in 10 mL of distilled water. The control treatment consisted of 10 mL of distilled water. Treatments were prepared on the morning of each study day, filled into a syringe by a research officer who had no involvement in data analysis, and administered at a temperature of ~30 °C.

### 2.4. Study Protocol

Each female participant was studied on 3 occasions, receiving either 300 mg or 600 mg of quinine or control, and studies were performed during the follicular phase of the menstrual cycle (i.e., days 1–8) to minimise any potential confounding effect on the secretion of gut hormones or gastric emptying [27]. Each male participant was studied on 2 occasions, receiving either 600 mg of quinine or control. Study treatments were administered in a randomised, double-blind fashion. To minimise any carry-over effect, study visits were separated by at least 3, and up to 7, days. Participants were instructed to refrain from vigorous physical activity and alcohol consumption for 24 h prior to each study and were provided with a standardised meal (beef lasagne; total energy content: 602 kcal; McCain Food, Wendouree, VIC, Australia) to be consumed between 6.30–7 p.m. on the evening before each study visit. The next morning, the participant attended the Clinical Research Facility (Adelaide Medical School, University of Adelaide) at 8.15 a.m. as well as after fasting overnight from both solids and liquids (with the exception of water) after 7 p.m. and from water after 6.30 a.m.

Upon arrival, the participant was intubated with a manometric catheter (Dentsleeve International, Mui Scientific, Mississauga, Ontario, Canada; total length: 100 cm; external diameter: 3.5 mm), which was positioned as described previously [3,28]. An infusion port, situated ~14.5 cm beyond the pylorus, was used for the infusion of quinine.

Once the catheter was positioned correctly (within 53 ± 6 min), an intravenous cannula was placed into a forearm vein. After the occurrence of phase III (short phase of motor activity characterised by high-amplitude, high-frequency contractions) of the fasting motility pattern, during phase I (a period of motor quiescence), a baseline blood sample and visual analogue scale (VAS) ratings (to assess GI symptoms) were collected, and QHCl or the control was delivered intraduodenally (at t = −31 min) within 1 min. The catheter was then removed. At t = −1 min, participants consumed, within 1 min, 350 mL of a mixed-nutrient drink (Resource plus, Nestle, Sydney, NSW, Australia; 325 mL, 500 kcal, 74 g carbohydrates, including maltodextrin and sucrose, 18 g protein and 15 g fat, plus 25 mL water), which included 100 mg of sodium acetate-1-^13^C for measurement of gastric emptying by breath test. Blood samples for measurement of hormones, VAS ratings and breath samples were collected at regular time intervals. At t = 120 min, the i.v. cannula was removed, after which each participant was provided with a light lunch and was then free to leave the laboratory.

### 2.5. Measurements

#### 2.5.1. Plasma Glucose and Hormone Analyses

Blood samples were collected into ice-chilled tubes containing tripotassium ethylenediaminetetraacetic acid. Plasma was obtained by centrifuging samples at ~1832 g force for 15 min at 4 °C within 15 min of collection and stored at −80 °C until subsequent analysis.

Plasma glucose concentrations (mmol/L) were measured by the glucose oxidase method, using a glucose analyser (YSI 2300 Stat Plus, Yellow Springs Instruments, Yellow Springs, OH, USA).

Plasma insulin concentrations (mU/L) were measured by a commercial ELISA immunoassay (Mercodia, Uppsala, Sweden). The sensitivity of the assay was 1.0 mU/L, and intra- and inter-assay CVs were 2.9% and 11.6%, respectively.

Plasma *C*-peptide concentrations (pmol/L) were measured by ELISA immunoassay (Mercodia, Bayswater, Victoria, Australia). The sensitivity of the assay was 15 pmol/L, and intra- and inter-assay CVs were 8.6% and 5.0%, respectively.

Plasma total GLP-1 concentrations (pmol/L) were measured by radioimmunoassay (Millipore, Billerica, MA, USA). The minimum detectable concentration was 3 pmol/L, and intra- and inter-assay CVs were 7% and 11%, respectively.

Plasma GIP concentrations (pmol/L) were measured by an ‘in-house’ radioimmunoassay using an antiserum against human GIP (Peninsula Laboratories, San Carlos, CA, USA), described in detail previously [29]. The minimum detectable concentration was 2 pmol/L, and intra- and inter-assay CVs were 3.4% and 8.5%, respectively.

Plasma CCK concentrations (pmol/L) were measured by an ‘in-house’ radioimmunoassay, described in detail previously [30]. The minimum detectable concentration was 0.1 pmol/L, and intra- and inter-assay CVs were 5% and 15%, respectively.

#### 2.5.2. Gastric Emptying

Gastric emptying was measured by breath test using sodium acetate-1-^13^C [31], as described previously [21].

### 2.6. Data and Statistical Analysis

Statistical analysis was performed using SPSS software (version 27.0; IBM, Chicago, IL, USA).

Fasting concentrations of plasma glucose and hormones (*C*-peptide, insulin, GLP-1, GIP and CCK) across study days were compared using repeated-measures two-way ANOVAs with treatment (QHCl-600, QHCl-300 (in females only), control), sex and their interaction as factors.

The effects of QHCl alone (i.e., before the consumption of the drink), and in response to the drink, on plasma glucose and hormone concentrations were evaluated in each group using repeated-measures two-way ANOVAs with treatment (QHCl-600, QHCl-300, control), time (i.e., t = −31, −21, −11 and −1 min; or t = −1, 10, 20, 30, 45, 60, 90 and 120 min, as relevant) and their interaction as fixed factors. Peak plasma glucose, time to peak glucose and concentrations at t = 30 min (a measure of the early rise) were analysed using repeated measures one-way ANOVA with treatment (QHCl-600, QHCl-300 and control) as a factor. The insulin secretory response was estimated as the ratio of change in insulin to that of glucose at t = 30 min, represented as ∆AUC_insulin-1 to 30_/∆AUC_glucose-1 to 30_ [32]. Insulin sensitivity was expressed as 1/fasting insulin. The oral disposition index was then calculated as ∆AUC_insulin-1 to 30_/∆AUC_glucose-1 to 30_ × 1/fasting insulin [33] and analysed using repeated measures one-way ANOVA with treatment (QHCl-600, QHCl-300 and control) as a factor. The plasma *C*-peptide to plasma glucose (*C*-peptide/glucose) ratio, a marker of beta cell function [34], was calculated for QHCl alone (AUC_−31 to −1_) and in response to the drink (AUC_−1 to 120_), and analysed using repeated measures one-way ANOVAs with treatment (QHCl-600, QHCl-300 and control) as a factor. The effect of quinine on gastric emptying was analysed using repeated measures two-way ANOVA with treatment (QHCl-600, QHCl-300 and control), time (i.e., t = −1, 10, 20, 30, 45, 60, 90 and 120 min) and their interaction as factors. Subject effects were accounted for by assuming that outcomes were correlated equally across multiple visits. Sphericity was evaluated by Mauchly’s test and, when violated, the adjusted Greenhouse–Geisser *p* value was reported. Post hoc comparisons, with *p* values adjusted for multiple comparisons by Bonferroni’s correction, were performed, where ANOVAs showed significant main effects or interactions. The normality and variance assumptions of ANOVA analyses were assessed via residual plots and acceptable for all models.

To compare the magnitude of effects between females and males (effects of sex) of QHCl-600 on plasma glucose, hormones, the *C*-peptide/glucose ratio and gastric emptying relative to control, data for glucose and hormones were expressed as AUCs (calculated using the trapezoidal rule) for the response to QHCl alone (AUC_−31 to −1_) and the drink (AUC_−1 to 120_), and AUC_−1 to 120_ only for gastric emptying data. Data were then analysed using mixed effects maximum likelihood models with treatments (QHCl-600, control), sex and the treatment-by-sex interaction as fixed factors, and random subject effects assuming an unstructured covariance matrix to account for the repeated visits per subject. To compare the magnitude of effects on the lowering of peak glucose, time to peak glucose and glucose concentrations at t = 30 min, values for QHCl-600 were subtracted from control values, and the data were then analysed using independent samples *t*-test.

Relations between plasma glucose (AUC_−1 to 30 min_, glucose at t = 30 min, peak glucose), hormones (AUC_−31 to −1 min_, concentration at t = −1 min, AUC_−1 to 30 min_) and gastric emptying (AUC_−1 to 30 min_) were evaluated using within-subject correlation analysis [35].

Differences were considered statistically significant at *p* ≤ 0.05. All data are reported as means ± SEM.

## 3. Results

All study treatments were well tolerated, and all participants completed the study visits. Oral QHCl detection thresholds were 0.15 ± 0.06 mmol/L in males and 0.10 ± 0.02 mmol/L in females, with no difference between the groups.

### 3.1. Basal Plasma Glucose and Hormone Concentrations

There were no differences in baseline concentrations of plasma glucose, *C*-peptide/glucose ratio or hormones between study days in either females or males, or between groups (Table 1).

### 3.2. Plasma Glucose Concentrations

#### 3.2.1. Dose-Related Effects in Females

**Figure 2:**
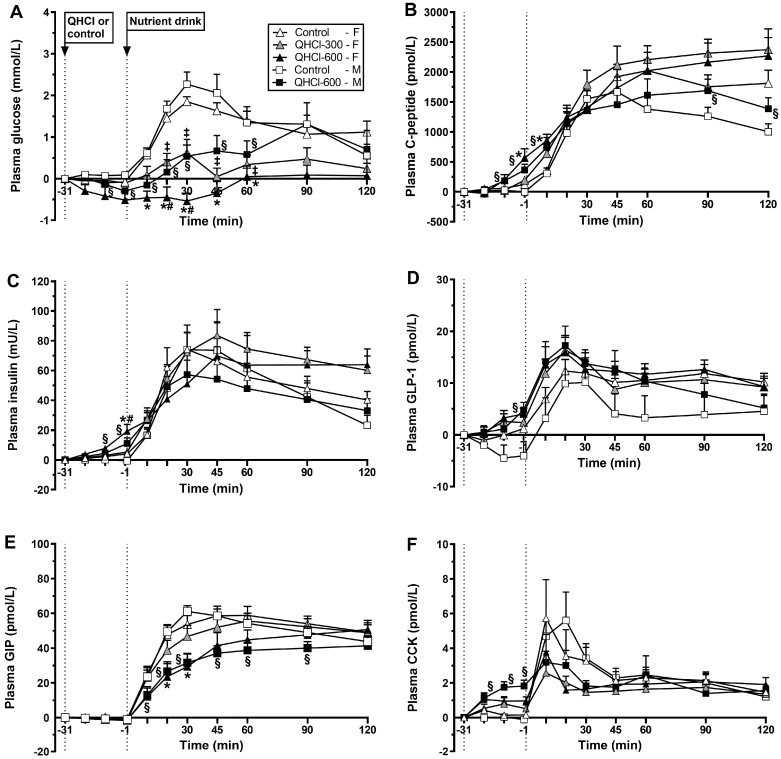
Plasma concentrations of (**A**) glucose, (**B**) *C*-peptide, (**C**) insulin, (**D**) glucagon-like peptide-1 (GLP-1), (**E**) glucose-dependent insulinotropic polypeptide (GIP) and (**F**) cholecystokinin (CCK) after the intraduodenal administration of quinine hydrochloride (QHCl), in doses of 300 (QHCl-300; in females only) and 600 mg (QHCl-600), or control (t = −31 to −1 min), and after a mixed-nutrient drink (t = −1–120 min) in 13 healthy women and 13 healthy men. Data were analysed using repeated measures two-way ANOVAs with treatment (QHCl-600, QHCl-300 and control), time and their interaction as factors, for the effects of QHCl alone and in response to the drink. (**A**) In females: * QHCl-600 significantly different from control (*p* < 0.05); # QHCl-600 significantly different from QHCl-300 (*p* < 0.05); ‡ QHCl-300 significantly different from control (*p* < 0.05); in males: § QHCl-600 significantly different from control (*p* < 0.05). (**B**) In females: * QHCl-600 significantly different from control (*p* < 0.05); in males: § QHCl-600 significantly different from control (*p* < 0.05). (**C**) In females: * QHCl-600 significantly different from control (*p* < 0.05); # QHCl-600 significantly different from QHCl-300 (*p* < 0.05); in males: § QHCl-600 significantly different from control (*p* < 0.05). (**D**) In males: § QHCl-600 significantly different from control (*p* < 0.05). (**E**) In females: * QHCl-600 significantly different from control (*p* < 0.05); in males: § QHCl-600 significantly different from control (*p* < 0.05). (**F**) In males: § QHCl-600 significantly different from control (*p* < 0.05). Data are means ± SEMs and changes from baseline.

Response to QHCl alone. There was no effect of treatment or time on plasma glucose, although mean concentrations were lower after QHCl-600 (Figure 2A).

Response to the drink. There was a treatment × time interaction for plasma glucose (*p* = 0.001) (Figure 2A). QHCl-600 reduced plasma glucose between t = 10–60 min compared with control between t = 20–30 min compared to QHCl-300 (all *p* < 0.05). QHCl-300 reduced plasma glucose between t = 20 and 60 min compared to the control (all *p* < 0.05). Moreover, glucose increased compared with baseline from t = 20 to 120 min after control (*p* < 0.05) and at t = 30 min after QHCl-300 (*p* < 0.05) but not after QHCl-600.

**Figure 3:**
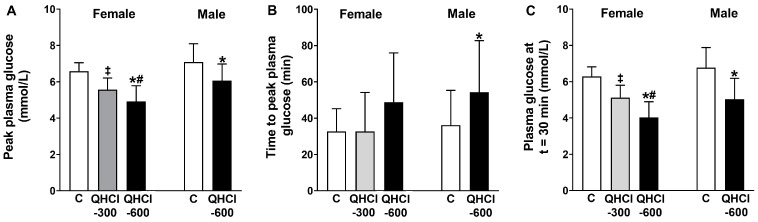
(**A**) Peak plasma glucose concentration, (**B**) time to peak plasma glucose and (**C**) plasma glucose concentration at t = 30 min after a mixed-nutrient drink, which was consumed 30 min after the intraduodenal administration of quinine hydrochloride (QHCl), in doses of 300 (QHCl-300; in females only) and 600 mg (QHCl-600), or control in 13 healthy women and 13 healthy men. Data were analysed using one-way ANOVA with treatment (QHCl-600, QHCl-300 and control) as a factor. * QHCl-600 significantly different from respective control (*p* < 0.05); # QHCl-600 significantly different from QHCl-300 (*p* < 0.05); ‡ QHCl-300 significantly different from control (*p* < 0.05). Data are means ± SEMs.

There were effects of treatment on peak plasma glucose (*p* = 0.001), the time to peak glucose (*p* = 0.043) and plasma glucose at t = 30 min (*p* = 0.001) (Figure 3). QHCl-600 reduced peak glucose compared with control (*p* = 0.001) and QHCl-300 (*p* = 0.020), and QHCl-300 compared to the control (*p* = 0.001). QHCl-600 also reduced plasma glucose at t = 30 min compared to the control (*p* = 0.001) and QHCl-300 (*p* = 0.002), and QHCl-300 compared to the control (*p* = 0.001).

#### 3.2.2. Effects in Males

Response to QHCl alone. There was no effect of treatment, but there was an effect of time (*p* = 0.001), on plasma glucose (Figure 2A). QHCl-600 reduced plasma glucose at t = −1 min compared to baseline (*p* < 0.05).

Response to the drink. There was a treatment × time interaction for plasma glucose (*p* = 0.008) (Figure 2A). QHCl-600 reduced glucose between t = 10 and 60 min compared to the control (*p* < 0.05). After the consumption of the drink, glucose increased compared to baseline, from t = 10 to 90 min after the control (*p* < 0.05), and at t = 30, 45 and 90 min after QHCl-600 (*p* < 0.05).

QHCl-600 also reduced peak glucose (*p* = 0.005), increased the time to peak glucose (*p* = 0.034), and reduced glucose at t = 30 min (*p* = 0.001), compared to the control (Figure 3).

#### 3.2.3. Comparison between Males and Females

Response to QHCl alone. There was no difference in the magnitude of glucose lowering by QHCl-600 between females and males (Table 2).

**Figure 4:**
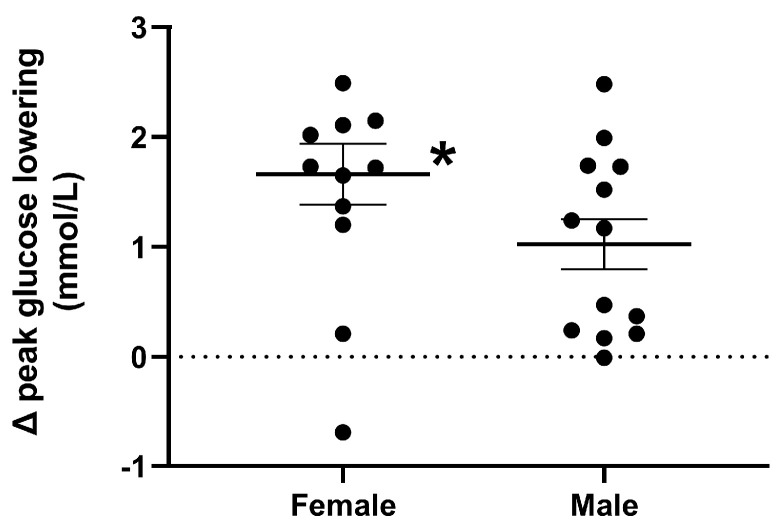
Magnitude of peak plasma glucose lowering, in response to a mixed-nutrient drink that was consumed 30 min after the intraduodenal administration of quinine hydrochloride (QHCl), in a dose of 600 mg (QHCl-600) relative to control, in 13 healthy women and 13 healthy men. To compare the magnitude of glucose lowering, values obtained after QHCl-600 were subtracted from those after control in each group, and the data was then analysed using one-way ANOVA with sex as a factor. * Females significantly different from males (*p* < 0.05). Data are means ± SEMs.

Response to the drink. The magnitude of the overall lowering of plasma glucose by QHCl-600 was greater in females than in males (*p* = 0.041) (Table 2). The magnitude of the lowering of peak glucose was greater in females than in males (*p* = 0.044) (Figure 4).

### 3.3. Plasma C-Peptide Concentrations

#### 3.3.1. Dose-Related Effects in Females

Response to QHCl alone. There was a treatment × time interaction for plasma *C*-peptide (*p* = 0.005) (Figure 2B). QHCl-600 stimulated *C*-peptide at t = −1 min compared to the control (*p* = 0.049) but not QHCl-300, which did not have an effect. QHCl-600 and QHCl-300, but not the control, stimulated *C*-peptide compared to baseline at t = −1 min (*p* < 0.05). There was no effect of treatment on the *C*-peptide/glucose ratio.

Response to the drink. There was a treatment × time interaction for plasma *C*-peptide (*p* = 0.029) (Figure 2B). QHCl-600 increased *C*-peptide at t = 10 min compared to the control (*p* = 0.003) but not QHCl-300, which did not have an effect. After each of the control, QHCl-300 and QHCl-600, *C*-peptide increased compared to baseline (*p* < 0.05). There was an effect of treatment on the *C*-peptide/glucose ratio (*p* = 001), which was greater after QHCl-600 and QHCl-300 compared to the control (both *p* = 0.001).

#### 3.3.2. Effects in Males

Response to QHCl alone. There was a treatment × time interaction for *C*-peptide (*p* = 0.006) (Figure 2B); QHCl-600 increased *C*-peptide between t = −11 and −1 min compared to the control (both *p* < 0.05). QHCl-600, but not the control, stimulated *C*-peptide compared to baseline from t = −11 to −1 min (*p* < 0.05). There was an effect of treatment on the *C*-peptide/glucose ratio (*p* = 0.006), which was greater after QHCl-600 compared to the control (*p* = 0.006).

Response to the drink. There was a treatment × time interaction for plasma *C*-peptide (*p* = 0.036) (Figure 2B); QHCl-600 increased *C*-peptide at t = 10 min and between t = 90 and 120 min compared to the control (all *p* < 0.05). On both the control and QHCl-600 days, *C*-peptide increased compared to the baseline (*p* < 0.05). There was an effect of treatment on the *C*-peptide to glucose ratio (*p* = 0.001), which was greater after QHCl-600 compared to the control (*p* = 0.001).

#### 3.3.3. Comparison between Males and Females

There was no difference in the stimulation of *C*-peptide by QHCl-600 between females and males, either in response to QHCl alone or the drink (Table 2).

### 3.4. Plasma Insulin Concentrations

#### 3.4.1. Dose-Related Effects in Females

Response to QHCl alone. There was a treatment × time interaction for plasma insulin (*p* = 0.004) (Figure 2C). QHCl-600 stimulated insulin at t = −1 min compared to the control (*p* = 0.029) and QHCl-300 (*p* = 0.026), while QHCl-300 did not have a significant effect. QHCl-600 and QHCl-300, but not the control, stimulated insulin, QHCl-600 from t = −21 to −1 min (*p* < 0.05), and QHCl-300 at t = −1 min (*p* < 0.01).

Response to the drink. There was no effect of treatment on plasma insulin concentrations (Figure 2C). After each, control, QHCl-300 and QHCl-600, insulin increased from t = 10 to 120 min (*p* < 0.05).

There was no effect of treatment on either the early insulin secretary response or insulin sensitivity (Table 3). There was a trend for an effect of treatment on the oral disposition index (*p* = 0.068) (Table 3).

#### 3.4.2. Effects in Males

Response to QHCl alone. There was a treatment × time interaction for plasma insulin (*p* = 0.009) (Figure 2C). QHCl-600 increased insulin between t = −11 and −1 min compared to the control (both *p* < 0.05). QHCl-600, but not the control, stimulated insulin compared to baseline at t = −1 min (*p* < 0.05).

Response to the drink. There was no effect of treatment on plasma insulin (Figure 2C). On both control and QHCl-600 days, insulin increased compared to baseline (*p* < 0.05).

There was no effect of treatment on the early insulin secretory response, insulin sensitivity nor the oral disposition index (Table 3).

#### 3.4.3. Comparison between Males and Females

There were no differences in the magnitude of the stimulation of insulin, the early insulin secretary response nor the oral disposition index by QHCl-600 between females and males (Table 2).

### 3.5. Plasma GLP-1 Concentrations

#### 3.5.1. Dose-Related Effects in Females

Response to QHCl alone. There were effects of treatment (*p* = 0.023) and time (*p* = 0.001) on plasma GLP-1 (Figure 2D). QHCl-600 stimulated GLP-1 compared to the control (*p* = 0.034) but not to QHCl-300, while QHCl-300 did not have an effect. QHCl-600, but neither QHCl-300 nor control, stimulated GLP-1 compared to baseline at t = −1 min (*p* < 0.05).

Response to the drink. There were effects of treatment (*p* = 0.044) and time (*p* = 0.001) on plasma GLP-1 (Figure 2D). QHCl-600 increased GLP-1 compared to the control (*p* = 0.031) but not QHCl-300, while QHCl-300 did not have an effect. After each of the control, QHCl-300 and QHCl-600, GLP-1 increased compared to baseline, from t = 20 to 120 min (*p* < 0.05).

#### 3.5.2. Effects in Males

Response to QHCl alone. There was a treatment × time interaction for plasma GLP-1 (*p* = 0.019) (Figure 2D). QHCl-600 increased GLP-1 at t = −1 min compared to the control (*p* = 0.009). QHCl-600, but not control, stimulated GLP-1 compared to baseline at t = −1 min (*p* = 0.05).

Response to the drink. There was a non-significant effect of treatment (*p* = 0.078) and an effect of time (*p* < 0.05) on plasma GLP-1 (Figure 2D). QHCl-600 tended to increase GLP-1 compared to the control (*p* = 0.078). On both control and QHCl-600 days, GLP-1 increased compared to baseline (*p* < 0.05).

#### 3.5.3. Comparison between Males and Females

There were no differences in the magnitude of the stimulation of GLP-1 by QHCl-600 between females and males (Table 2).

### 3.6. Plasma GIP Concentrations

#### 3.6.1. Dose-Related Effects in Females

Response to QHCl alone. There was no effect of treatment, or time, on plasma GIP (Figure 2E).

Response to the drink. There was a treatment × time interaction for plasma GIP (*p* = 0.023) (Figure 2E). QHCl-600 reduced GIP between t = 20 and 30 min compared to the control (*p* < 0.05) but not QHCl-300, while QHCl-300 did not have a significant effect. The control, QHCl-300 and QHCl-600 each increased GIP compared to baseline, from t = 10 to 120 min (*p* < 0.05).

#### 3.6.2. Effects in Males

Response to QHCl alone. There was no effect of treatment, or time, on plasma GIP (Figure 2E).

Response to the drink. There was a treatment × time interaction for GIP (*p* = 0.001) (Figure 2E). GIP was lower after QHCl-600 compared to the control between t = 10 and 90 min (*p* < 0.05). After the control and QHCl-600, GIP increased compared to baseline (*p* < 0.05).

#### 3.6.3. Comparison between Males and Females

There were no differences in the magnitude of the stimulation of GIP by QHCl-600 between females and males, either in response to QHCl alone or the drink (Table 2).

### 3.7. Plasma CCK Concentrations

#### 3.7.1. Dose-Related Effects in Females

Response to QHCl alone. There was a trend for a treatment × time interaction (*p* = 0.074) for plasma CCK (Figure 2F). QHCl-600 stimulated CCK from t = −21 to −1 min (*p* < 0.05) compared to the control, and at t = −1 min compared to QHCl-300 (*p* = 0.01). QHCl-600, but not QHCl-300 or the control, stimulated CCK compared to baseline from t = −21 to −1 min (*p* < 0.05).

Response to the drink. There was no effect of treatment, but there was an effect of time (*p* = 0.01), on plasma CCK (Figure 2F). After each of the control, QHCl-300 and QHCl-600, CCK increased compared to baseline (all *p* < 0.05).

#### 3.7.2. Effects in Males

Response to QHCl alone. There was a treatment × time interaction for plasma CCK (*p* = 0.001) (Figure 2F). QHCl-600 increased CCK between t = −21 and −1 min compared to the control (all *p* < 0.05). QHCl-600, but not the control, stimulated CCK compared with baseline from t = −21 to −1 min (*p* = 0.05).

Response to the drink. There was an effect of treatment on plasma CCK (*p* = 0.031) (Figure 2F). Plasma CCK was greater after the control than QHCl-600 (*p* = 0.031). On both the control and QHCl-600 days, CCK increased compared to baseline (*p* < 0.05).

#### 3.7.3. Comparison between Males and Females

There was no difference in the magnitude of the stimulation of CCK by QHCl-600 between females and males after the drink, but the response to QHCl alone was non-significantly greater in males than in females (*p* = 0.079) (Table 2).

### 3.8. Gastric Emptying

#### 3.8.1. Dose-Related Effects in Females

**Figure 5:**
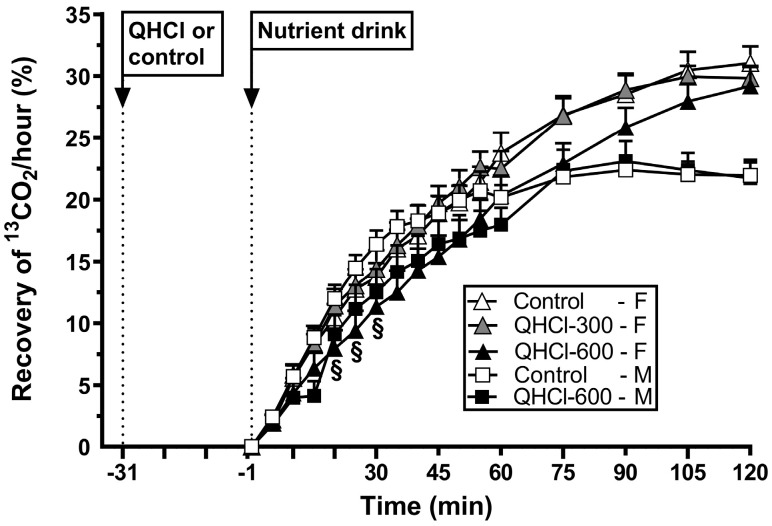
Gastric emptying of a mixed-nutrient drink, measured by ^13^C-acetate breath test, consumed 30 min after the intraduodenal administration of quinine hydrochloride (QHCl), in doses of 300 (QHCl-300; in females only) and 600 mg (QHCl-600), or control, in 13 healthy women and 13 healthy men. Data were analysed using two-way ANOVAs with treatment (QHCl-600, QHCl-300 and control), time and their interaction as factors. § QHCl-600 significantly different from control in males (*p* < 0.05). Data are means ± SEMs.

There was an effect of treatment on gastric emptying (*p* = 0.021) (Figure 5). QHCl-600 slowed gastric emptying compared to QHCl-300 (*p* = 0.027), but not the control, while QHCl-300 did not have an effect.

#### 3.8.2. Effects in Males

There was a treatment × time interaction for gastric emptying (*p* = 0.048) (Figure 5). QHCl-600 slowed emptying between t = 20 and 30 min compared to the control (all *p* < 0.05).

#### 3.8.3. Comparison between Males and Females

There was no difference in the magnitude of the slowing of gastric emptying by QHCl-600 between females and males (Table 2).

### 3.9. Relations between Plasma Glucose, Insulin, C-Peptide, GLP-1, CCK and Gastric Emptying

In females, there were inverse correlations between plasma glucose AUC_−1 to 30 min_ with insulin AUC_−31 to −1 min_ (*r* = −0.366, *p* = 0.021), insulin at t = −1 min (*r* = −0.397, *p* = 0.012), *C*-peptide at t = −1 min (*r* = −0.357, *p* = 0.032), GLP-1 AUC_−31 to −1 min_ (*r* = −0.376, *p* = 0.023), GLP-1 at t = −1 min (*r* = −0.340, *p* = 0.042), and GLP-1 AUC_−1 to 30 min_ (*r* = −0.331, *p* = 0.048), and non-significant inverse correlations between peak plasma glucose and GLP-1 AUC_−31 to −1 min_ (*r* = −0.282, *p* = 0.095) and CCK AUC_−31 to −1 min_ (*r* = −0.299, *p* = 0.064). There were inverse correlations between gastric emptying AUC_−1–30 min_ and GLP-1 at t = −1 min (*r* = −0.408, *p* = 0.013), CCK AUC_−31 to −1 min_ (*r* = −0.469, *p* = 0.005) and CCK at t = −1 min (*r* = −0.564, *p* = 0.001).

In males, there were inverse correlations between plasma glucose AUC_−1 to 30 min_ with insulin AUC_−31 to −1 min_ (*r* = −0.391, *p* = 0.047), insulin at t = −1 min (*r* = −0.483, *p* = 0.012), *C*-peptide at t = −1 min (*r* = −0.416, *p* = 0.043) and CCK at t = −1 min (*r* = −0.516, *p* = 0.002). There were direct correlations between plasma glucose AUC_−1 to 30 min_ (*r* = 0.404, *p* < 0.001) and plasma glucose at t = 30 min (*r* = −0.546, *p* = 0.005) with gastric emptying AUC_−1–30 min_.

## 4. Discussion

Our study evaluated the dose-related effects of ID QHCl on GI functions that contribute to blood glucose regulation, including glucoregulatory hormones and gastric emptying, in healthy women, and whether the effects of QHCl are influenced by sex. Key findings are that in females, quinine stimulated insulin, *C*-peptide, GLP-1 and CCK, particularly before the meal; slowed gastric emptying; dose-dependently reduced plasma glucose; and delayed the rise in glucose after the meal; and that both glucose lowering after the meal and the *C*-peptide/glucose ratio were greater in women than in men. Thus, the glucose-lowering effect of quinine appears to be sex-dependent.

We reported recently that in healthy men, quinine, in a dose of 600 mg, potently stimulates plasma insulin, *C*-peptide, GLP-1 and CCK; slows gastric emptying; and lowers the plasma glucose response to a carbohydrate-rich drink [20,21]. Furthermore, the ID administration of quinine stimulated gut and pancreatic hormones and pyloric pressures more than IG administration [20], and the stimulation of insulin by ID quinine was greater in women than in men, which is associated with a reduction in baseline glucose levels [20]. We have now established that quinine has potent, and dose-related, effects to lower the glucose response to a carbohydrate-rich, nutrient drink in healthy, young females, and that the magnitude of glucose lowering is greater in females than in males.

The glucose response to a meal is determined by glucoregulatory hormones, including GLP-1, GIP and insulin, as well as the rate of gastric emptying [36], which itself is regulated by both GLP-1 and CCK [37]. Indeed, in both females and males, plasma glucose during the first 30 min post-drink, expressed as AUC_−1 to 30 min_ and reflecting the main response to the drink, correlated inversely with plasma insulin and *C*-peptide, as well as GLP-1 (in females) or CCK (in males) responses before the drink. Thus, these hormones, stimulated by quinine *before* the drink, appeared to influence the glucose response *to* the drink. While a relationship between plasma glucose and gastric emptying was only evident in males, in females, gastric emptying correlated inversely with concentrations of both GLP-1 and CCK before the drink.

In females, the 600 mg dose of quinine abolished the rise in plasma glucose after the drink, which likely reflects the interplay between a number of factors, including the slowing of gastric emptying, mediated, in part, by GLP-1 and CCK [36,37] and, potentially, the direct effect of quinine on gut smooth muscle cells [38], the stimulation of insulin, triggered by GLP-1 in a glucose-dependent fashion and, possibly, the direct action of quinine on pancreatic beta cells, as well as a direct effect of GLP-1 on nerve endings in the portal vein [39]. In contrast, GIP does not appear to have played a role, given that levels were lower after quinine, most likely because of the greater slowing of gastric emptying. While the 300 mg dose also reduced plasma glucose and peak glucose markedly after the drink, its effects were less than that of the 600 mg dose. The latter also occurred in the absence of slowing of gastric emptying or differences in plasma GLP-1 or CCK. However, the 300 mg dose had a modest effect in stimulating plasma insulin and *C*-peptide immediately before the drink. This suggests that while this dose of quinine was insufficient to reach a threshold to stimulate GLP-1 and CCK associated with slowing of gastric emptying, it had the capacity to stimulate insulin immediately before the drink, possibly by direct action on pancreatic beta cells, which is likely responsible for the observed glucose lowering.

Our study provides evidence that the glucose-lowering effect of quinine may be sex-dependent. Thus, quinine reduced postprandial glucose more in females than males without differences in the magnitude of GI hormone secretion or gastric emptying. We did not adjust the quinine doses used for differences in body weight between males and females, but this is unlikely to account for the observed difference in blood glucose lowering, given that none of the other measured parameters were affected. We did not measure glucagon, but found no difference in the effect of quinine on glucagon between males and females in our recent study [20]. The finding that the *C*-peptide/glucose ratio was greater in females than males suggests that a given amount of *C*-peptide had a greater glucose-lowering capacity, indicative of a greater sensitivity to the glucose-lowering effect of insulin. Similarly, the greater glucose lowering in females may have reflected an enhanced sensitivity to the glucose-lowering actions of other glucoregulatory hormones, e.g., GLP-1, by quinine. Alterations in the sensitivity to GI hormones have been observed in other populations, including an augmented sensitivity to CCK in patients with functional dyspepsia [40], or an attenuated sensitivity to the satiating effects of CCK in people with obesity [41], although whether these latter findings are sex-specific remains to be established. Further studies are indicated to clarify this issue.

The observed patterns of GLP-1, GIP and CCK are of interest. Quinine alone stimulated both CCK and GLP-1 modestly during the first 30 min, as reported [20], but did not affect GIP. GLP-1 continued to rise after the drink until mean levels after quinine were higher than those after control. In contrast, GIP and CCK levels only rose slightly and then plateaued after QHCl-600, while yielding more elevated concentrations on the control day, in both males and females. While we had anticipated GIP and CCK to rise in a fashion similar to that of GLP-1, it is possible that the slowing of gastric emptying, by QHCl-600, reduced the exposure of the proximal small intestine, the primary location of GIP and CCK secretion, to the nutrients contained in the drink, compromising the post-drink stimulation of both hormones. In contrast, GIP and CCK stimulation in response to the drink on the control day presumably reflected prompt duodenal nutrient exposure. Plasma GLP-1 concentrations, on the other hand, did not appear to be affected by the slowing of gastric emptying, probably because GLP-1 is primarily secreted from the distal small intestine, and quinine that escaped absorption proximally may have contributed to ongoing GLP-1 stimulation.

The limitations of our study should be noted. While the number of participants is relatively small, the number was derived using power calculations based on our previous studies [21,22]. Due to the small number of participants within each sequence group (*n* = 4), we were unable to adjust the analyses for sequence. We did not measure plasma quinine concentrations, which may have provided insights into whether the observed effects of quinine reflect the interaction of circulating quinine with smooth muscle or pancreatic beta cells rather than the activation of bitter receptors on enteroendocrine cells and the involvement of gut-related mechanisms. In this context, it is important to recognise, as discussed, that the intravenous administration of quinine, as used in the treatment of malaria, stimulates insulin [12] and can cause hypoglycaemia [11], indicating that a direct effect on the pancreas is likely to contribute to glucose lowering. We did not measure plasma concentrations of ghrelin or motilin, which may be stimulated by bitter substances [42,43]. While both hormones accelerate gastric emptying [44,45], ghrelin is suppressed after a meal [1]; thus, they are unlikely to be relevant to the observed slowing of gastric emptying by quinine. We administered quinine intraduodenally since ID has greater effects than IG administration [20], presumably reflecting the significance of the activation of small intestinal bitter receptors by quinine. Because quinine has an extremely bitter taste, it would be impossible to assess the oral effects of the dose used in our study (or even smaller doses). After drink ingestion, our study design by definition did not allow for the distinction of the effects of quinine from those of the drink. However, the primary purpose was to evaluate the effects of quinine on gut and glucoregulatory hormones and the postprandial glycaemic response.

## 5. Conclusions

We conclude that quinine modulates glucoregulatory functions, including the stimulation of insulin, GLP-1 and CCK, as well as the slowing of gastric emptying associated with the lowering of the glycaemic response to a nutrient drink, which was dose-related in females. While the glucose-lowering effect of quinine, in a dose of 300 mg, was not associated with detectable gut-related effects, the lower dose of quinine is sufficient for marked glucose lowering, which has potential therapeutic implications. Our study provides evidence of sex differences in the effects of quinine to lower postprandial glucose, indicative of the potential for personalised approaches to glucose lowering. Taken together, our observations in healthy people (that is, with normal glycaemic control) have implications for the potential administration of quinine (or quinine derivates, perhaps particularly those that do not induce hypoglycaemia) to reduce postprandial glycaemic excursions as a specific therapeutic target in individuals with type 2 diabetes or impaired glucose tolerance, particularly in females. This warrants further investigation.

## Figures and Tables

**Table 1:** Fasting plasma concentrations of glucose, glucoregulatory and gut hormones, and plasma *C*-peptide to glucose ratio across study days in females and males.

	Female			Male	
	Control	QHCl-300	QHCl-600	Control	QHCl-600
Plasma glucose, mmol/L	4.4 ± 0.1	4.5 ± 0.1	4.5 ± 0.1	4.5 ± 0.1	4.4 ± 0.1
Plasma *C*-peptide, pmol/L	420 ± 54	470 ± 73	436 ± 52	308 ± 65	320 ± 59
Plasma *C*-peptide/glucose, pmol/mmol	100 ± 14	105 ± 18	99 ± 14	68 ± 12	73 ± 11
Plasma insulin, mU/L	2.4 ± 0.5	2.4 ± 0.4	2.5 ± 0.4	2.3 ± 0.6	2.4 ± 0.5
Plasma GLP-1, pmol/L	13 ± 1	13 ± 2	14 ± 1	22 ± 2	21 ± 1
Plasma GIP, pmol/L	13.6 ± 1.6	13.2 ± 1.6	13.8 ± 1.8	16.7 ± 1.3	15.8 ± 1.3
Plasma CCK, pmol/L	0.8 ± 0.1	0.9 ± 0.1	0.7 ± 0.1	1.2 ± 0.1	0.9 ± 0.1

Data are means ± SEMs; *n* = 13 in each group, except for plasma *C*-peptide and GLP-1 (*n* = 12). QHCl-300, quinine-hydrochloride in a dose of 300 mg; QHCl-600, quinine-hydrochloride in a dose of 600 mg; GLP-1, glucagon-like peptide-1; GIP, glucose-dependent insulinotropic polypeptide; CCK, cholecystokinin.

**Table 2:** Comparative effects of quinine alone (t = −31 to −1 min), overall (t = −1 to 120 min) and early (t = −1 to 30 min) on plasma glucose, glucoregulatory hormone responses and *C*-peptide/glucose ratio to a mixed-nutrient drink, and overall (t = −1 to 120 min) and early (t = −1 to 30 min) gastric emptying of the drink, ingested 30 min after the intraduodenal administration of quinine in a dose of 600 mg, or control, in females and males.

	Female		Male	
	Control	QHCl-600	Control	QHCl-600
**Plasma glucose**				
AUC_−31 to −1 min_, mmol/L × min	130 ± 2	127 ± 4	136 ± 1	131 ± 2.6
AUC_−1 to 120 min_, mmol/L × min	636 ± 17	485 ± 27 *	650 ± 16	579 ± 26
AUC_−1 to 30 min_, mmol/L × min	116 ± 3	81 ± 4	121 ± 2	93 ± 4
**Plasma *C*-peptide**				
AUC_−31 to −1 min_, pmol/L × min	12,799 ± 642	15,714 ± 1458	11,831 ± 651	15,391 ± 1454
AUC_−1 to 120 min_, pmol/L × min	105,502 ± 11,611	242,883 ± 21,243	184,632 ± 11,734	202,656 ± 21,185
AUC_−1 to 30 min_, pmol/L × min	28,088 ± 2608	31,625 ± 3631	26,177 ± 2632	28,753 ± 3617
**Plasma *C*-peptide/glucose ratio**				
AUC_−31 to −1 min_, pmol/mmol × min	107 ± 16	142 ± 20	66 ± 13	103 ± 16
AUC_−1 to 120 min_, pmol/mmol × min	178 ± 17	536 ± 57 *	261 ± 25	339 ± 33
**Plasma insulin**				
AUC_−31 to −1 min_, mU/L × min	127 ± 32	290 ± 58	98 ± 21	195 ± 44
AUC_−1 to 120 min_, mU/L × min	6182 ± 606	6801 ± 1214	5946 ± 1502	5207 ± 1135
AUC_−1 to 30 min_, mU/L × min	1124 ± 231	844 ± 227	999 ± 158	959 ± 239
**Plasma GLP-1**				
AUC_−31 to −1 min_, pmol/L × min	450 ± 29	525 ± 30	514 ± 30	595 ± 30
AUC_−1 to 120 min_, pmol/L × min	2684 ± 192	2938 ± 213	2985 ± 196	3280 ± 211
AUC_−1 to 30 min_, pmol/L × min	483 ± 53	576 ± 57	611 ± 54	701 ± 56
**Plasma GIP**				
AUC_−31 to −1 min_, pmol/L × min	446 ± 16	428 ± 16	439 ± 16	433 ± 16
AUC_−1 to 120 min_, pmol/L × min	7672 ± 431	6263 ± 508	7343 ± 434	5670 ± 506
AUC_−1 to 30 min_, pmol/L × min	1428 ± 101	934 ± 142	1497 ± 102	1005 ± 142
**Plasma CCK**				
AUC_−31 to −1 min_, pmol/L × min	31 ± 3	48 ± 7	36 ± 5	65 ± 8
AUC_−1 to 120 min_, pmol/L × min	365 ± 55	303 ± 32	423 ± 87	316 ± 39
AUC_−1 to 30 min_, pmol/L × min	97 ± 29	58 ± 7	121 ± 25	74 ± 14
**Gastric emptying**				
AUC_−1 to 120 min_, % × min	2484 ± 118	2167 ± 140	2030 ± 115	2021 ± 138
AUC_−1 to 30 min_, % × min	232 ± 21	178 ± 35	245 ± 20	198 ± 32

Data are means ± SEMs; *n* = 13 in each group, except for plasma *C*-peptide, GLP-1 and gastric emptying (*n* = 12). AUC, area under the curve; QHCl-600, quinine-hydrochloride in a dose of 600 mg; GLP-1, glucagon-like peptide-1; GIP, glucose-dependent insulinotropic polypeptide; CCK, cholecystokinin. * Significantly different from male (*p* < 0.05).

**Table 3:** Early insulin secretary response, insulin sensitivity and oral disposition index to a mixed-nutrient drink, ingested 30 min after the intraduodenal administration of quinine, in doses of 300 mg and 600 mg, or control, in females and males.

	Female			Male	
	Control	QHCl-300	QHCl-600	Control	QHCl-600
Early insulin secretory response (mU/mmol × min) ∆AUC_insulin-1 to 30_/∆AUC_glucose-1 to 30_	8.9 ± 1.8	10.8 ± 1.7	13.5 ± 3.3	7.7 ± 1.7	10.0 ± 2.2
Insulin sensitivity (mU/L^−1^) 1/fasting insulin	0.6 ± 0.1	0.6 ± 0.1	0.5 ± 0.1	0.5 ± 0.1	0.7 ± 0.2
Oral disposition index (mM^−1^ × min) ∆AUC_insulin-1 to 30_/∆AUC_glucose-1 to 30_ × 1/fasting insulin	3.9 ± 0.5	5.1 ± 0.9	6.2 ± 1.0 *	2.9 ± 0.5	6.6 ± 2.0 *

Data are means ± SEMs; *n* = 13 in each group, except for plasma *C*-peptide, GLP-1 and gastric emptying (*n* = 12). AUC, area under the curve; QHCl-600, quinine-hydrochloride in a dose of 600 mg; GLP-1, glucagon-like peptide-1; GIP, glucose-dependent insulinotropic polypeptide; CCK, cholecystokinin. * Significantly different from male (*p* < 0.05).

## Data Availability

The data presented in this study are available upon request from the corresponding author. The data are not publicly available due to privacy reasons.
